# Habitat suitability and driving factors of the endangered medicinal plant *Sinopodophyllum hexandrum* under climate and land use change

**DOI:** 10.3389/fpls.2026.1809149

**Published:** 2026-06-03

**Authors:** Gong-Han Tu, Li Liu, Fei Chen, Shao-Yang Xi, Xu-Dong Guo, Zhi-Xian Jing, Ling Jin

**Affiliations:** 1 College of Pharmacy, Gansu University of Chinese Medicine, Lanzhou, China; 2 Gansu Pharmaceutical Industry Innovation Research Institute, Lanzhou, China; 3 State Key Laboratory for Quality Ensurance and Sustainable Use of Dao-di Herbs, Beijing, China; 4 Northwest Collaborative Innovation Center for Traditional Chinese Medicine Co-Constructed by Gansu Province & Ministry of Education of the People's Republic of China, Lanzhou, China

**Keywords:** climate change, conservation planning, geographical detector, land use, MaxEnt model, *Sinopodophyllum hexandrum*

## Abstract

**Introduction:**

Climate change and land-use change pose significant threats to the survival of endangered medicinal plants. This study focuses on the endangered medicinal plant *Sinopodophyllum hexandrum*.

**Methods:**

Using 268 distribution records and an optimized MaxEnt model (RM = 4.0, FC=LQHPT), together with an OptimalParameters Geographical Detector (OPGD), the current and future habitat suitability,driving mechanisms, and the impact of land use change under variousclimate scenarios (SSP126, SSP370, SSP585) were systematically evaluated.

**Results:**

The results indicate that: (1) The current suitable habitat area for *S. hexandrum* is 1.1608 × 10⁶ km², primarily distributed in Sichuan, Tibet, and Gansu along the eastern edge of the Qinghai-Tibet Plateau. High suitability areas are concentrated at altitudes between 2800–3500 m. (2) Future climate warming is projected to promote the northwestward expansion of suitable areas. Under the SSP585 scenario, the suitable habitat area is expected to increase to 1.8770 × 10⁶ km² by the 2090s, representing a 61.70% increase from the current area, with the habitat centroid shifting by 333.74 km. (3) Altitude (contribution rate of 34.5%, q = 0.245), minimum temperature of the coldest month (26.4%), and annual precipitation (20.7%) are the dominant factors influencing distribution. Interactions among environmental factors significantly enhance explanatory power, with the strongest synergistic effect observed for bio12 ∩ elevation (q = 0.685). (4) High-risk areas (as defined by the OPGD Risk Detector) cover 5.30 × 10⁴ km², with 75.3% located outside existing nature reserves. (5) Grassland (4.979 × 10⁵ km²) and forest land (4.731 × 10⁵ km²) are the primary carrier ecosystems, with moderately suitable grassland areas projected to increase under future climate scenarios.

**Discussion:**

This study reveals the strict ecological requirements of *S. hexandrum* for high-altitude, low-temperature, and moderate‑precipitation environments, as well as the synergistic effects of hydrothermal coupling on its distribution. The findings provide a scientific basis for conservation planning, the designation of priority conservation areas, and climate‑adaptive management of endangered medicinal plants.

## 1 Introduction

Medicinal plants play a pivotal role in global healthcare systems and the development of Traditional Chinese Medicine, serving as a source of bioactive compounds widely used in pharmaceuticals, herbal medicines, and for maintaining healthy lifestyles (Djouwoug et al., 2021; Borcă et al., 2024). Endangered medicinal plants are not only repositories of phytochemical diversity with potential for novel drug discovery but also serve as crucial indicator species for ecosystem health and resilience under global environmental change (Howes et al., 2020; Theodoridis et al., 2023). The concept of “One Health” further underscores the strategic importance of medicinal plant conservation, highlighting the intrinsic connections between human, animal, and environmental health (Asigbaase et al., 2023; McMahon Barry et al., 2025).

Of approximately 300,000 plant species globally, 21,000 possess medicinal value. The World Health Organization (WHO) predicts that 60% of the global population and 80% of the population in developing countries rely on herbal remedies for their health (Pandita et al., 2021; Patni et al., 2021). However, human activities, including climate change and land-use change pose severe threats to the survival and sustainable utilization of medicinal plants, impacting their supply and biodiversity (Mykhailenko et al., 2025; Zaman et al., 2025). Climate change is among the most critical threats to current biodiversity (Parmesan and Yohe, 2003), leading not only to shifts in the geographical distribution of medicinal plants, loss of suitable habitats, and potential species extinction, but also to alterations their medicinal efficacy and quality by affecting the synthesis of secondary metabolites (Cahyaningsih et al., 2021; Ebi et al., 2021; Xia et al., 2022; Yao et al., 2022; Sun et al., 2023; Jangpangi et al., 2025). Concurrently, land-use change, particularly urbanization (Xiao et al., 2024), agricultural intensification (de la Riva et al., 2023), mining activities (Zhang et al., 2024b), and deforestation (Kemppinen et al., 2024), exerts continuous pressure on medicinal plant populations regarding abundance, diversity, and distribution through habitat destruction, landscape fragmentation, and localized microclimate alterations, threatening global ecosystem functions.

The MaxEnt model, a widely used ecological niche modeling approach, accurately predicts the potential suitable distribution areas of endangered medicinal plants by integrating species distribution records with environmental variables. It is particularly useful for cases with sparse distribution data, effectively identifying key environmental factors and quantifying their contributions, thus providing a spatial basis for delineating conservation priority areas and guiding *ex-situ* conservation efforts (Xia et al., 2022; Xu et al., 2024). Since default MaxEnt parameters may cause overfitting and reduce model transferability, species-specific tuning of the regularization multiplier (RM) and feature combination (FC) was performed to improve model reliability. The Geographical Detector, a statistical method based on spatial variance analysis, quantifies the impact of key driving factors on species distribution and reveals the interaction mechanisms among different environmental factors, thereby leading to a deeper understanding of species’ ecological requirements (Shi et al., 2023a). The combination of MaxEnt and the Geographical Detector has been successfully applied to evaluate the ecological suitability of various endangered medicinal plants, including species in the Saussurea genus (Zhao et al., 2024), Coptis genus (Li et al., 2020), and *Bletilla striata* (Mu et al., 2025), providing crucial technical support for developing conservation strategies, optimizing cultivation and introduction, and achieving sustainable resource utilization.

The endangered medicinal plant *S. hexandrum* is an important precursor for the synthesis of anti-cancer drugs such as etoposide due to its high content of podophyllotoxin (PPT). Its fruits and extracts also contain various bioactive components like flavonoids and polysaccharides, exhibiting significant anti-tumor, anti-inflammatory, and antioxidant activities, holding a significant position in modern medicine and traditional Tibetan medicine (Liu et al., 2021, 2023). However, due to multiple pressures including over-harvesting, habitat degradation, climate change, and altered land use patterns, the wild populations of *S. hexandrum* have been declining, leading to its classification as endangered on the IUCN Red List (Zhang et al., 2025c). *S. hexandrum* is primarily distributed in the high-altitude alpine regions of the Himalayas, between 2400–4500 meters, with strict ecological requirements for temperature, precipitation, and light. Furthermore, its complex seed dormancy, low natural regeneration rate, and long growth cycle have hindered the development of mature artificial cultivation techniques, making it difficult to meet market demands (Zhao et al., 2023). Under the intensifying pressures of climate change and human activities, accurately assessing the habitat suitability of *S. hexandrum*, predicting changes in its potential distribution, and elucidating the environmental driving mechanisms are crucial for developing scientific conservation plans, delineating priority conservation areas, and guiding sustainable utilization. To address these questions, the study employed an optimized MaxEnt model for habitat suitability predictions and future distribution projections, and an Optimal Parameters Geographical Detector (OPGD) to identify the dominant environmental factors and their interactions.

This study, based on distribution data of *S. hexandrum*, utilizes an optimized MaxEnt model and an Optimal Parameters Geographical Detector (OPGD) to predict the potential distribution of *S. hexandrum* in China under current and future climate scenarios, its ecological niche dynamics, climate adaptive responses, and driving factors. The specific research objectives are: (1) What is the potential suitable distribution of *S. hexandrum* under current and future climate conditions? (2) How will climate change affect its future distribution and spatial pattern? (3) How do environmental factors influence the spatial distribution of *S. hexandrum* habitats? (4) How does land use change threaten the suitable habitats of *S. hexandrum*? Addressing these questions will provide a theoretical basis for the strategic planning of *S. hexandrum* cultivation and introduction. Furthermore, the research findings will offer scientific evidence and practical guidance for the conservation of endangered medicinal plant resources and the establishment of nature reserves, ensuring the protection of its ecological and economic value amidst the challenges posed by climate and land use change.

## 2 Materials and methods

### 2.1 Acquisition and processing of distribution data for *S. hexandrum*

**Figure 1:**
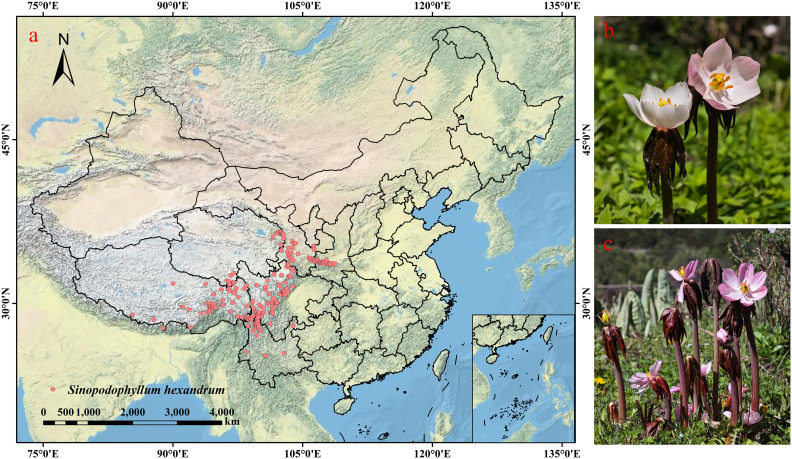
Occurrence points **(A)** and photographs **(B, C)** of *S. hexandrum*. Photographs were taken at the Shika Snow Mountain, Shangri-La by Liu Yifeng, used with permission.

Geographical distribution point data for *S. hexandrum* were obtained from the Global Biodiversity Information Facility (GBIF, https://www.gbif.org, accessed June 20, 2025), iNaturalist (https://www.inaturalist.org, accessed June 20, 2025), field surveys, and relevant literature (Liu et al., 2014, 2015). To mitigate potential overfitting caused by sampling bias in the MaxEnt model and to reduce the impact of redundant data on prediction accuracy, data were filtered using ENMTools 5.26 software (http://enmtools.blogspot.com/). According to the resolution of the ecological factor data used in the model, only one species distribution point was retained within each 5 km × 5 km grid. Distribution points outside China were also excluded. Ultimately, 268 distribution records for *S. hexandrum* were obtained and saved in.csv format for MaxEnt model construction (**Figure 1**).

### 2.2 Environmental variable selection and data processing

**Figure 2:**
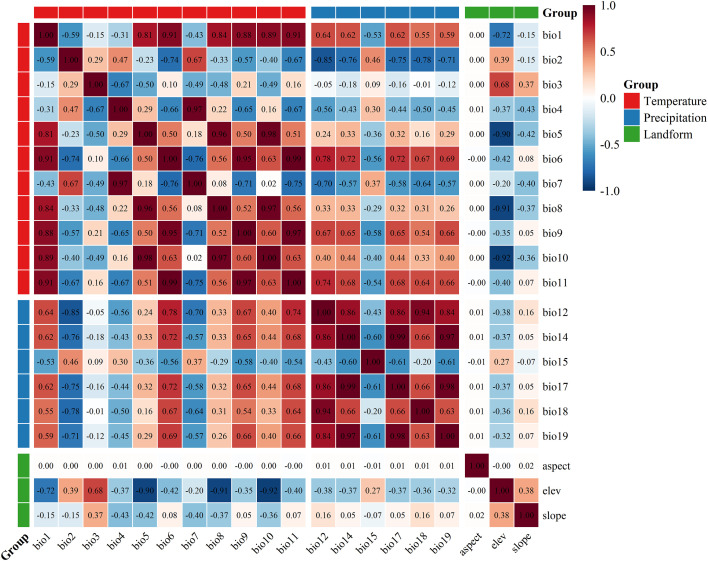
Correlation matrix of environmental variables.

Current climate data (1970-2000) and future climate data for 2041-2060 (2050s), 2061-2080 (2070s), and 2081-2100 (2090s) were downloaded from WorldClim (v2.1, https://www.worldclim.org) and encompassed 19 bioclimatic variables (Bio1-19) and 3 topographical factors (elev, aspect, slope), all at a 2.5′ resolution. Bio1-Bio10 are temperature-related variables, and Bio11-Bio19 are precipitation-related variables (Huang et al., 2025). Future projections were conducted under three Shared Socioeconomic Pathways (SSPs) from the Coupled Model Intercomparison Project Phase 6 (CMIP6): SSP126 represents low vulnerability, low mitigation pressure, and low radiative forcing; SSP370 represents a combination of high social vulnerability and relatively high anthropogenic radiative forcing; and SSP585 represents a high forcing scenario (Wu et al., 2019). Atmospheric CO_2_ concentration is a primary driver of global temperature; therefore, CMIP6 was selected because of its high accuracy in predicting changes in CO_2_ concentration and its applicability as a general climate system model for most terrestrial plants. In this framework, anthropogenic emissions of CO_2_ and other greenhouse gases are considered the main drivers of climate change (Lv et al., 2025). This study used data from the Beijing Climate Center Climate System Model (BCC-CSM2-MR) which is recognized for its excellent performance in Chinese climate projections (Xi et al., 2025). To avoid model overfitting due to multicollinearity among environmental variables, variables with correlation coefficients (|r| ≥ 0.8) and low contribution rates were removed, based on preliminary MaxEnt model runs assessing variable contribution and permutation importance. Additionally, variables relevant to the actual growth requirements of *S. hexandrum* were retained (Shi et al., 2023b). Consequently, nine environmental variables were selected (**Supplementary Table 1**), with the correlation coefficient matrix presented in **Figure 2**.

### 2.3 Species distribution model construction

MaxEnt models are sensitive to sampling bias and prone to overfitting when default parameters are used. Therefore, relying on default settings may yield unreliable predictions, particularly for species with limited occurrence records. The regularization multiplier (RM) and feature combination (FC) are critical factors influencing the complexity of MaxEnt models (Qin et al., 2023). To identify the optimal balance between model fit and complexity, this study employed the ENMeval package (R platform 4.3.3) to optimize MaxEnt model parameters (Muscarella et al., 2014). RM values ranged from 0.5 to 4.0, with increments of 0.5, resulting in eight levels. Six feature combination (FC) types were considered: L (linear), LQ (linear + quadratic), LQH (linear + quadratic + hinge), H (hinge), LQHP (linear + quadratic + hinge + product), and LQHPT (linear + quadratic + hinge + product + threshold). Through cross-combination, 48 parameter schemes were generated, and for each combination, the Akaike information criterion corrected for small sample sizes (AICc) was calculated. AICc evaluates model performance while penalizing unnecessary complexity; lower values indicate a more parsimonious model. AICc = 0 was selected as the optimal parameter set for MaxEnt modeling (Wu et al., 2024). Subsequently, the MaxEnt (v3.4.1) model was used to predict the potential suitable distribution of *S. hexandrum*. The distribution point data (.csv) and environmental data (.asc) were imported into the software. The “clipping” method was selected, and the output format was set to logistic. Twenty-five percent of the distribution data were designated for model testing, with the remaining 75% used for training. The optimized RM and FC values were applied, and the model was run 10 times with default settings for other parameters.

Model accuracy was assessed using the omission rate and the area under the Receiver Operating Characteristic (ROC) curve (AUC). A closer agreement between the test omission rate and the theoretical omission rate indicates higher model accuracy. AUC values range from 0 to 1; values below 0.6 suggest model failure, 0.6< AUC ≤ 0.7 indicate poor prediction, 0.7< AUC ≤ 0.8 suggest general prediction, 0.8< AUC ≤ 0.9 indicate accurate prediction, and 0.9< AUC ≤ 1.0 signifies highly accurate prediction (Zhao et al., 2024). The final model selection was guided not only by high AUC but also by low omission rates and parsimony (i.e., the optimized parameter set), ensuring that the predicted distribution is both statistically robust and ecologically meaningful.

The MaxEnt model automatically outputs the average of 10 replicate runs, with results presented as a logistic suitability index ranging from 0 to 1, where higher values indicate greater habitat suitability for *S. hexandrum*. Using a manual classification method based on the suitability index, suitable areas were categorized into four levels: unsuitable (P< 0.1), low suitability (0.1 ≤ P< 0.3), moderate suitability (0.3 ≤ P< 0.5), and high suitability (P ≥ 0.5). The area of each suitability class was then calculated (Qiu et al., 2025). To visually represent the changes in potential suitable areas for *S. hexandrum* in China under different climate scenarios, ArcGIS software was used to calculate the proportion and area of different suitability classes from raster layer attribute tables. Python-based SDMTools was used to analyze areas of stability, expansion, and contraction, as well as centroid shifts in suitable areas under different climate scenarios. Finally, maps illustrating changes in potential suitable areas and centroid migration routes were generated.

### 2.4 Multivariate environmental similarity surface and most dissimilar variable

The MESS and MoD methods were used to assess the degree of climate anomaly between current and future periods and to identify key factors contributing to changes in potential suitable habitats. MESS quantifies the similarity between climate conditions at a specific point in the future and the reference climate conditions, expressed as a similarity index (S). Lower S values indicate greater divergence from reference climate conditions, with S = 100 representing no difference. Negative S values indicate that at least one climate variable exceeds the reference range, with more negative values signifying a greater climate anomaly. MoD identifies the variable with the lowest similarity at a given point, potentially highlighting the key factors driving distribution changes (Shi et al., 2024). By identifying and analyzing these variables, we can gain a clearer understanding of potential barriers to the expansion of suitable habitats for *S. hexandrum* can be gained, providing valuable insights for future conservation and cultivation efforts. In this study, bioclimatic variables from the current suitable habitat were used as the reference layer. This analysis was conducted using the “density.tools. Novel” tool within the maxent.jar file (Elith et al., 2010).

### 2.5 Niche measurement

This study quantitatively analyzed the ecological niche of *S. hexandrum* under different temporal periods and climate models. The “ecospat” R package (v4.5.1) was used for ecological niche analysis, examining the relationship between the species and its habitat, and its changes under different climate scenarios (Broennimann et al., 2012; Zhang et al., 2025a). Principal Component Analysis (PCA-env) was first applied to reduce the dimensionality of bioclimatic variables, producing a two-dimensional environmental space to simplify the data structure and highlight the primary influences of environmental variables on species distribution. Within ecospat, niche overlap was measured using Hellinger’s I or Schoener’s D. This study employed Schoener’s D to quantify niche overlap under different environmental conditions, with values ranging from 0 (no overlap) to 1 (complete overlap). Values greater than 0.6 were considered significant overlap. Niche breadth was calculated using ENMTools (B1 for minimum breadth, B2 for maximum breadth), and bidirectional climate niche similarity tests (1000 replicates) were conducted to validate the reliability of the results.

### 2.6 Optimal parameters geographical detector model

The Geographical Detector (GD) is a statistical tool used to analyze spatial heterogeneity (Jinfeng and Chengdong, 2017). It integrates GIS, statistics, and mathematical modeling to efficiently identify primary influencing factors in spatial data and analyze interactions among these factors (He et al., 2023). Building upon the GD model, Song et al. introduced an improved model, the OPGD, designed to address the challenge of optimally discretizing continuous data (Song et al., 2020). Discretization methods employed included standard deviation (SD), natural breaks (NB), equal interval (EI), quantile (QU), and geometric interval (GI), which automatically classify spatial data into appropriate intervals. Compared to traditional regression models, the OPGD offers several advantages in analyzing spatial relationships and identifying the driving factors of geographical phenomena (Tan et al., 2025). The OPGD comprises four sub-models: Factor Detector, Interaction Detector, Ecological Detector, and Risk Detector. The Factor Detector is used to identifies the key environmental factors driving the spatial differentiation of potential habitat suitability for *S. hexandrum*. The Interaction Detector analyzes the interaction effects among different environmental factors. The Ecological Detector compares the significance of the influence of various environmental factors. The Risk Detector identifies high-risk areas for the distribution of *S. hexandrum*. Collectively, these detectors comprehensively reveal the mechanisms underlying the formation of *S. hexandrum’s* spatial distribution patterns, providing a scientific basis for future risk management and spatial decision-making.

#### 2.6.1 Factor detector

The Factor Detector is used to assess the explanatory power of environmental factor X on the spatial differentiation of *S. hexandrum’s* potential habitat suitability index Y, measured by the q-value (Equation 1):

(1) $$q=1-\frac{\sum_{h=1}^{L}N_{h}{\text{σ}}_{h}^{2}}{N\sigma^{2}}=1-\frac{SSW}{SST}$$

Where: h = 1,…, L represents the number of strata for variable Y or factor X; N_h_ and N are the number of units in stratum h and the entire region, respectively; and $\sigma_{h}^{2}$ and σ^2^ are the variances of Y values in stratum h and the entire region. The q-value ranges from 0 to 1, with larger values indicating stronger explanatory power of factor X on attribute Y.

#### 2.6.2 Interaction detector

The Interaction Detector identifies the interactions between different influence factors, evaluating whether the combined effect of factors X1 and X2 enhances or weakens the explanatory power on dependent variable Y. Interaction types are determined by comparing q(X1), q(X2), and q(X1 ∩ X2) (**Table 1**):

**Table 1:** Types of interaction effects between two independent variables on a dependent variable.

Judgments	Interaction types
*q*(X1∩X2)< Min(*q*(X1), *q*(X2))	Nonlinear weakening
Min(*q*(X1), *q*(X2))< *q*(X1∩X2)< Max(*q*(X1), *q*(X2))	Single-driver weakening
*q*(X1∩X2) > Max(*q*(X1), *q*(X2))	Double driver enhancement
*q*(X1∩X2) = *q*(X1) + *q*(X2)	Independence
*q*(X1∩X2) > *q*(X1) + *q*(X2)	Nonlinear enhancement

#### 2.6.3 Risk detector

The Risk Detector is used to determine if there are significant differences in attribute means between two sub-regions, employing the t-statistic (Equation 2) for testing:

(2) $$t=\frac{{\hat{Y}}_{h-1}-{\hat{Y}}_{h-2}}{{\left[\frac{\text{Var}\left(Y_{h-1}\right)}{n_{h-1}}+\frac{\text{Var}\left(Y_{h-2}\right)}{n_{h-2}}\right]}^{1/2}}$$

Where: ${\hat{\text{Y}}}_{\text{h}}$ represent the mean attribute values in sub-regions h; *n*_ℎ_ was the sample sizes in sub-regions h; and *Var* represent the variances. The null hypothesis *H*_0_ is ${\hat{\text{Y}}}_{\text{h}=1}={\hat{\text{Y}}}_{\text{h}=2}$. If *H*_0_ is rejected at a confidence level α, it indicates a significant difference in attribute means between the two sub-regions.

#### 2.6.4 Ecological detector

The Ecological Detector is used to compare the significant differences in the spatial influence of two factors, X1 and X2, on attribute Y, using the F-statistic (Equation 3) for testing:

(3) $$F=\frac{N_{X1}\left(N_{X2}-1\right)SSW_{X1}}{N_{X2}\left(N_{X1}-1\right)SSW_{X2}}$$

Where: NX1 and NX2 are the sample sizes for factors X1 and X2, respectively; and SSWX1 and SSWX2 are the sums of squares within strata formed by X1 and X2. The null hypothesis H_0_ is SSWX1 = SSWX2. If H_0_ is rejected at a significance level α, it indicates a significant difference in the spatial influence of the two factors on attribute Y.

The OPGD model was constructed in the R environment using the GD package. By exploring various discretization methods and class combinations, the optimal discretization scheme that maximized the q-value was automatically selected. The study area was divided into different strata based on the values and arrangements of independent variables, ensuring the maximum q-value influence for subsequent factor detection (Zhang et al., 2025b). The results and process of discretization optimization for different environmental factor variables are shown in **Supplementary Figure 1**.

### 2.7 Land use analysis of *S. hexandrum* suitable areas

The global land-use projection dataset for 2015–2100 was used as the source of land cover information (http://www.geosimulation.cn/Global-SSP-RCP-LUCC-Product.html), encompassing six land cover types: cropland, forest, grassland, urban, unused land, and water bodies (Chen et al., 2022). The dataset has a spatial resolution of approximately 1 km (0.01°). Quality assessment of the dataset indicated an average Kappa coefficient of 0.864 and an overall accuracy of 0.929 at the global scale, and a spatial consistency index of 0.102. For China, the corresponding indicators were 0.847, 0.886, and 0.137, respectively, all demonstrating good classification accuracy. This high-quality land-use dataset provides reliable information for evaluating environmental effects and studying climate change responses (Tu et al., 2025). Within the analysis framework, the predicted current and future climate scenario habitat suitability distribution results from the MaxEnt model were spatially overlaid with corresponding land-use scenario data. By calculating the areas of suitable habitats under different time points and climate scenario combinations, the impact of land-use change on species distribution was quantitatively assessed, and visualization techniques were employed to illustrate its spatiotemporal evolution.

## 3 Results

### 3.1 MaxEnt model optimization and result evaluation

**Figure 3:**
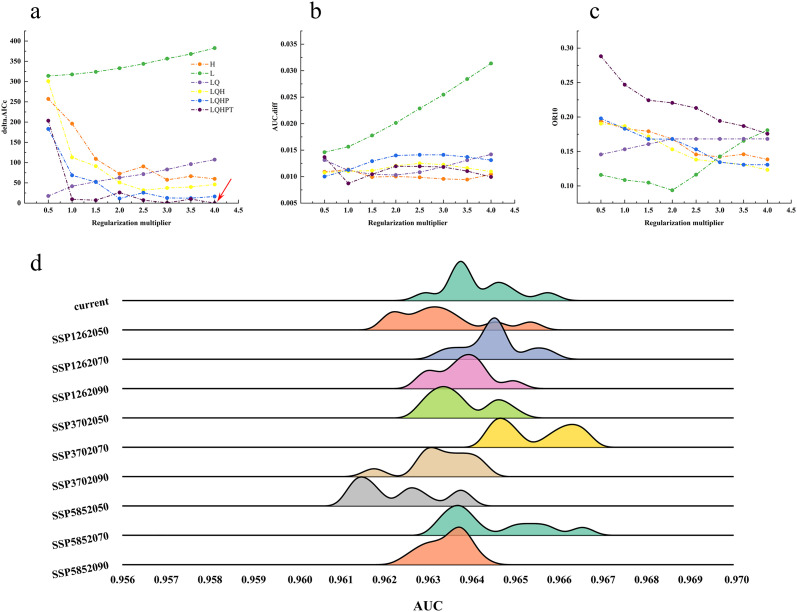
Model optimization and evaluation metrics: Delta AICc **(A)**, AUC difference **(B)**, Odds Ratio 10 **(C)**, and training AUC values under future climate scenarios **(D)**.

To achieve optimal species distribution predictions, the key parameters of the MaxEnt model were systematically optimized. The results indicated that the model with RM = 4.0 and FC=LQHPT exhibited the minimum AICc value (delta.AICc = 0), thus representing the optimal model according to the Akaike information criterion (**Figure 3A**). The optimized model showed lower AUC.diff and OR10 values compared to the default parameter model (**Figures 3B, C**), indicating a significant reduction in model complexity and an improvement in fit. The optimized parameter setting is therefore suitable for simulating the potential habitat of *S. hexandrum*. Under the optimal parameter settings, the average training AUC value from 10 MaxEnt model runs was 0.961 with a standard deviation of 0.007. The comprehensive average training AUC values across nine future climate scenarios exceeded 0.9, signifying highly accurate predictions (**Figure 3D**). Consequently, RM = 4 and FC = LQHPT were selected as the final parameter settings.

### 3.2 Dominant environmental variables and their response curves

Based on the MaxEnt model results, key environmental factors influencing the suitable habitat distribution of the target species were identified by analyzing variable contribution rates, permutation importance, and the relationship between environmental variables and species occurrence probability. The results indicated (**Table 2**) that altitude (elev, 34.5%), minimum temperature of the coldest month (bio6, 26.4%), and annual precipitation (bio12, 20.7%) were the dominant factors, with a cumulative contribution rate of 81.6%, highlighting them as core environmental elements controlling species distribution. Temperature seasonality (bio4) contributed 16.8%, also significantly impacting species distribution. In contrast, other environmental variables such as slope (slope), precipitation of the driest month (bio14), precipitation seasonality (bio15), slope aspect (aspect), and isothermality (bio3) had contribution rates below 1%, suggesting a limited direct influence on species distribution.

**Table 2:** Contribution rate and permutation importance value of each environmental variable.

Variable	Description	Percent contribution(%)	Permutation importance(%)
elev	Altitude	34.5	23.1
bio6	Min Temperature of Coldest Month	26.4	13.5
bio12	Annual Precipitation	20.7	23.9
bio4	Temperature Seasonality	16.8	17.1
slope	Slope gradient	0.4	0.1
bio14	Precipitation of Driest Month	0.4	18.4
bio15	Precipitation Seasonality	0.4	1.6
aspect	Slope aspect	0.2	0.3
bio3	Isothermality	0.1	1.8

Jackknife test results (**Supplementary Figure 2A**) demonstrated the importance of each environmental variable in the MaxEnt model and its standardized training gain. The performance order was “including all variables” > “excluding the variable” > “using only the variable,” emphasizing the higher model performance when all environmental variables are considered and highlighting the significance of their combined effects in accurately predicting species distribution. When using “only the variable,” altitude (elev), temperature seasonality (bio4), annual precipitation (bio12), and minimum temperature of the coldest month (bio6) exhibited high standardized gain values, underscoring their substantial contribution to model performance.

The response curves of the primary environmental variables revealed specific adaptation patterns of the species to environmental gradients, demonstrating significant non-linear relationships between key environmental factors and species occurrence probability (**Supplementary Figure 2B**). Moderate to high suitability (P > 0.3) for altitude was observed between 1620–4743 m, with suitability probability rapidly decreasing at excessively high or low altitudes. Temperature seasonality exhibited a suitable range of 473-819, indicating a requirement for moderate temperature fluctuations. Minimum temperature of the coldest month showed higher probability between -18 °C and -3 °C, peaking around -10 °C. Annual precipitation had higher suitability in the range of 486–973 mm, with a peak probability at 600–700 mm. These response curves, characterized by distinct environmental thresholds, align well with the concept of optimal environmental ranges in niche theory, indicating a clear ecological preference of this species for high-altitude, low-temperature, moderately changing temperatures, and moderately humid environmental conditions.

### 3.3 Potential distribution of *S. hexandrum* under current and future climate scenarios

**Figure 4:**
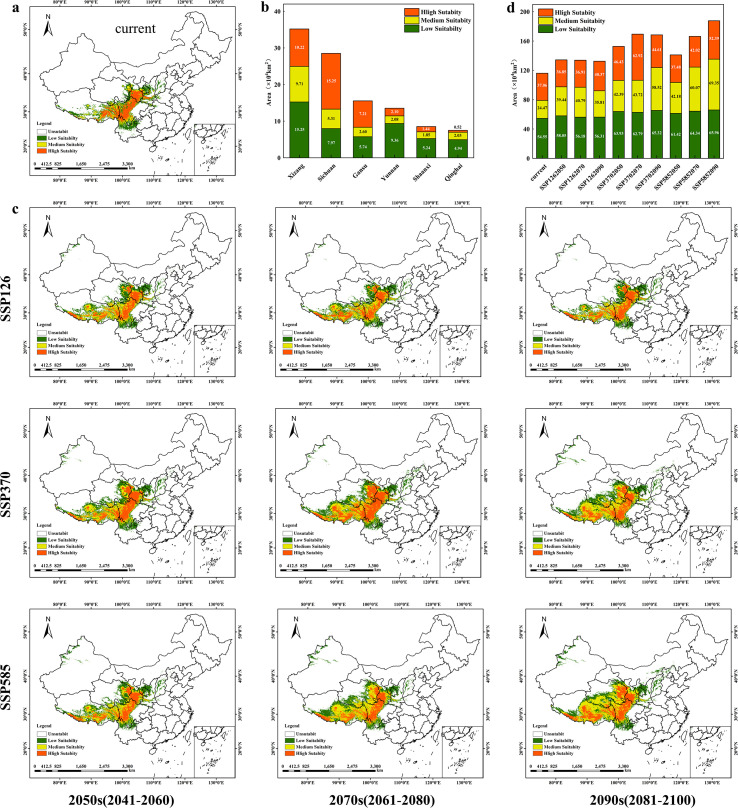
Potential distribution of *S. hexandrum* in China under current and future climate scenarios. **(A)** Potential distribution under the current climate scenario. **(B)** Area (×10^4^ km^2^) of the primary distribution regions by province under the current climate scenario. **(C)** Potential distribution under future climate scenarios. **(D)** Area (×10^4^ km^2^) of the potential distribution under future climate scenarios.

Species distribution modeling revealed a distinct spatial pattern of potential habitat suitability for *S. hexandrum* in China under current and future climate conditions (**Figure 4**). Under current climate conditions (**Figure 4A**), *S. hexandrum* is predominantly distributed in southwestern and central-western China. High suitability areas are mainly located in the eastern Qinghai-Tibet Plateau, including parts of Sichuan, Tibet, and contiguous areas of Gansu and Shaanxi provinces. The top provinces with high suitability areas (**Figure 4B**) are Sichuan (approximately 1.525 × 10^5^ km^2^), Tibet (approximately 1.022 × 10^5^ km^2^), and Gansu (approximately 7.21 × 10^4^ km^2^). Moderately suitable areas form transitional zones surrounding core suitable habitats, while low suitability areas extend into adjacent regions.

Future climate scenarios indicate (**Figures 4C, D**) a general expansion of suitable habitats with increasing global warming. Compared to the current suitable area (1.1608 × 10^6^ km^2^), habitat suitability area remains relatively stable under the SSP126 scenario, reaching 1.3249 × 10^6^ km^2^ by the 2090s. The SSP370 scenario shows a more pronounced increase in habitat area, reaching 1.6845 × 10^6^ km^2^ by the 2090s. The SSP585 scenario exhibits the most significant changes, with the total suitable area expanding to 1.8770 × 10^6^ km^2^ by the 2090s, a 61.70% increase from the current area. High suitability areas under SSP370 and SSP585 scenarios demonstrate a pattern of initial increase followed by a decrease, or continuous growth, respectively. Moderately and low suitability areas show expansion across all scenarios, suggesting that climate change provides new habitat opportunities for *S. hexandrum* to migrate to higher altitudes and latitudes, consistent with the general trend of upward migration observed in many alpine plants under warming conditions.

### 3.4 Changes in the spatial pattern and centroid migration of *S. hexandrum*’s potential distribution under future climate scenarios

**Figure 5:**
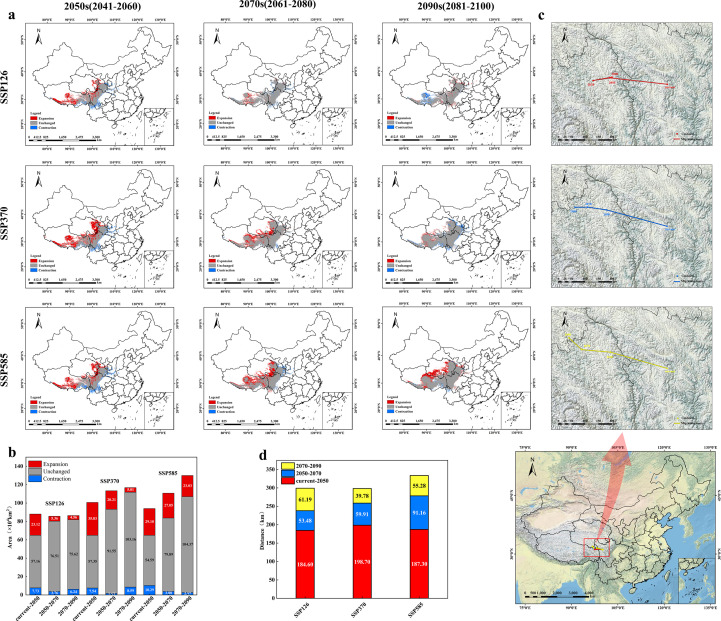
Changes in the potential distribution pattern and centroid shift of *S. hexandrum* under future climate scenarios. **(A)** Changes in potential distribution patterns under different climate scenarios. **(B)** Area of changes in the potential distribution patterns (×10^4^ km^2^) under different climate scenarios. **(C)** Shift paths of the potential distribution centroid under different climate scenarios. **(D)** Shift distances of the potential distribution centroid **(km)** under different climate scenarios.

The analysis of changes in the spatial pattern of *S. hexandrum*’s potential distribution under different future climate scenarios reveals distinct spatiotemporal variations (**Figure 5A**). Under the SSP126 scenario, the distribution range shows a gradual contraction, with expansion areas primarily concentrated on the southeastern edge of the Qinghai-Tibet Plateau and contraction areas occurring at the edges of the current distribution. The SSP370 scenario displays a more complex pattern, with expansion areas being more pronounced in the earlier periods, mainly extending into the hinterland of the Qinghai-Tibet Plateau and the northern regions of the Hengduan Mountains. The SSP585 scenario exhibits the most significant changes, with expansion areas continuously extending towards the northwest. Quantitative analysis indicates (**Figure 5B**) that the SSP370 scenario shows the greatest expansion potential, with an increase of 3.583 × 10^5^ km^2^ from the current period to the 2050s. The SSP585 scenario demonstrates a strong expansion trend in the first two periods, with expansion areas of 2.910 × 10^5^ km^2^ and 2.705 × 10^5^ km^2^, respectively. In contrast, the SSP126 scenario shows a smaller expansion amplitude, decreasing from 2.312 × 10^5^ km^2^ to 4.56 × 10^4^ km^2^.

The analysis of the centroid migration path of *S. hexandrum*’s potential distribution under different future climate scenarios reveals the species’ spatial response patterns to climate change (**Figure 5C**). Under the SSP126 scenario, the centroid migration path is relatively short, with minor shifts primarily in the northwest-southwest-northeast direction. The SSP370 scenario shows a clear northwestward migration trend with a longer and more directional migration path. The SSP585 scenario exhibits the most significant centroid migration, characterized by a clear northwestward long-distance migration. Quantitative analysis of centroid migration distances (**Figure 5D**) indicates that for the SSP126 scenario, migration distances over the three periods are 184.60 km, 53.48 km, and 61.19 km, respectively. For the SSP370 scenario, these distances are 198.70 km, 59.91 km, and 39.78 km, respectively. The SSP585 scenario shows the largest migration distances: 187.30 km, 91.16 km, and 55.28 km, respectively. This northwestward migration pattern reflects the adaptive response of *S. hexandrum* to varying intensities of climate change, indicating its spatial adaptive capacity to migrate to higher altitudes to cope with global warming.

### 3.5 MESS and MoD analysis for different future periods

**Figure 6:**
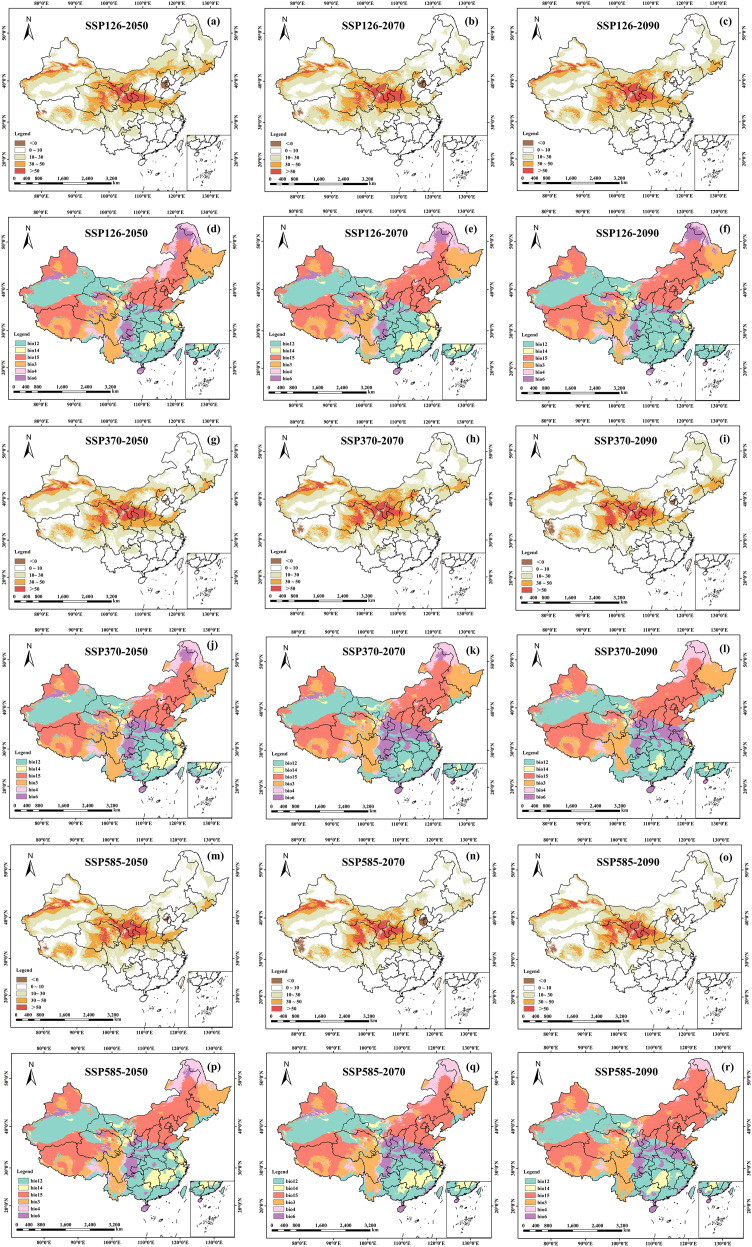
Results of the multivariate environmental similarity surface (MESS) and the most dissimilar variable (MoD) analyses for the distribution of *S. hexandrum* under different future climate scenarios. **(A-C, G-I, M-O)** MESS results, indicating environmental similarity to the current calibration area. **(D-F, J-L, P-R)** MoD results, identifying the variable contributing most to dissimilarity at each location.

Through MESS analysis, it was found that areas with high similarity in bioclimatic variables are primarily concentrated in northwestern and northern China (**Figures 6A-C, G-I, M-O**). In future periods, the bioclimatic conditions in these areas remain highly similar to the current period, suggesting that these regions may offer suitable habitats for *S. hexandrum* and possess strong adaptive potential to support its survival and expansion. However, western marginal areas, southwestern regions, and parts of the eastern coast exhibit significant differences in climate conditions compared to existing habitats, indicating potentially unfavorable conditions for *S. hexandrum*’s growth and distribution. Furthermore, MoD analysis revealed the bioclimatic variables with the greatest differences and lowest similarity between current and future periods (**Figures 6D-F, J-L, P-R**). Precipitation was identified as a key variable influencing *S. hexandrum*’s growth and adaptation, with the most significant differences observed in mean temperature of the wettest quarter (Bio8) and precipitation seasonality (Bio15). These variable differences may impact *S. hexandrum*’s growth cycle, reproductive capacity, and adaptability, consequently restricting its distribution range in China. This highlights the significant impact of climate change in terms of key variables on the species’ survival and expansion, particularly changes in precipitation patterns.

### 3.6 Ecological niche variation analysis for different future periods

**Figure 7:**
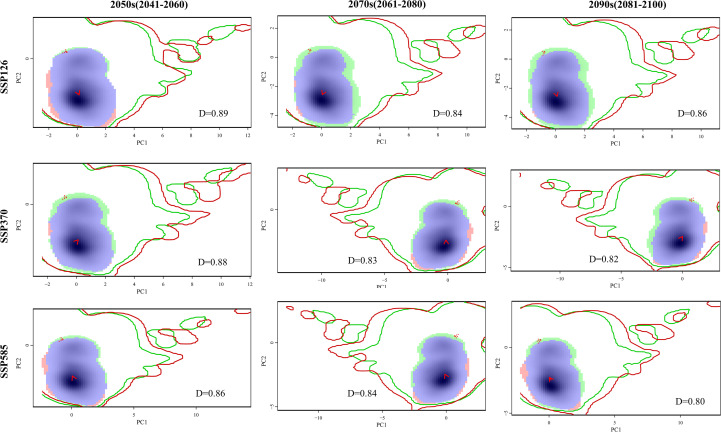
Niche changes of *S. hexandrum* under different future climate scenarios.

To investigate the spatiotemporal evolution of *S. hexandrum*’s ecological niche under future climate change scenarios, this study analyzed niche dynamics for three periods (2050s, 2070s, and 2090s) across three Shared Socioeconomic Pathways (SSP126, SSP370, and SSP585) (**Figure 7**). Principal Component Analysis indicated that the cumulative contribution of the first two principal components ranged between 65.22% and 66.49%, with PCA1 contributing 38.27%-39.52% and PCA2 contributing 26.81%-27.07%, effectively explaining the main environmental gradients of niche variation. Ecological niche overlap (D values) remained above 0.80 in all scenarios, suggesting that *S. hexandrum* will maintain a high level of ecological niche stability in the future. Under low emission scenarios (SSP126), D values were 0.89-0.84-0.86, with relatively stable niche centers. Under moderate to high emission scenarios (SSP370), D values decreased from 0.88 to 0.82, with a noticeable spatial shift in core suitable areas in the later period. Under high emission scenarios (SSP585), D values continuously decreased from 0.86 to 0.80, with significant spatial shifts in core suitable areas and considerable changes in the distribution patterns of expanding marginal areas. The results indicate that as emission intensity increases and time progresses, ecological niche overlap shows a declining trend. All scenarios exhibit significant changes in the 2070s, potentially representing a critical turning point for climate change impacts, thereby providing important basis for targeted conservation strategies.

### 3.7 Driving factor identification using optimal parameters geographical detector

#### 3.7.1 Factor detection and interaction detection analysis

**Figure 8:**
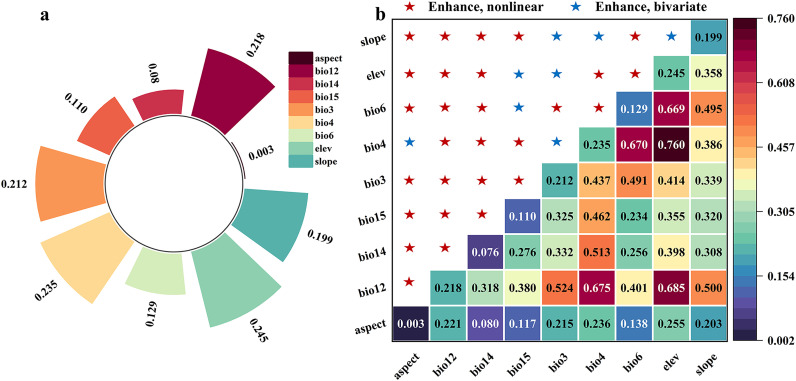
Analysis of single-factor and interaction effects on the spatial heterogeneity of *S. hexandrum* based on the OPGD model. **(A)** The q-statistic values of individual factors. **(B)** The interaction results between factors.

Factor detection results from the Optimal Parameters Geographical Detector model indicated (**Figure 8A**) that altitude (elev) exhibited the strongest explanatory power for the spatial distribution of *S. hexandrum* (q=0.245), followed by temperature seasonality (bio4, q=0.235), annual precipitation (bio12, q=0.218), isothermality (bio3, q=0.212), and slope (slope, q=0.199). Slope aspect had the weakest explanatory power (q=0.003). This suggests that altitude, temperature seasonality, and annual precipitation are the dominant factors influencing its distribution. Interaction detection results (**Figure 8B**) revealed that all factors exhibited enhancement effects, primarily nonlinear enhancement. Combinations such as bio12 ∩ elev (q=0.685), bio12 ∩ bio4 (q=0.675), bio4 ∩ bio6 (q=0.670), and bio6 ∩ elev (q=0.669) showed significantly higher explanatory power than any single factor. This indicates that the spatial distribution of *S. hexandrum* is primarily regulated by the synergistic effects of hydrothermal conditions, with topographic factors indirectly influencing the distribution pattern by altering local hydrothermal environments, reflecting the species’ comprehensive adaptation to complex environmental conditions.

#### 3.7.2 Risk detection analysis

**Figure 9:**
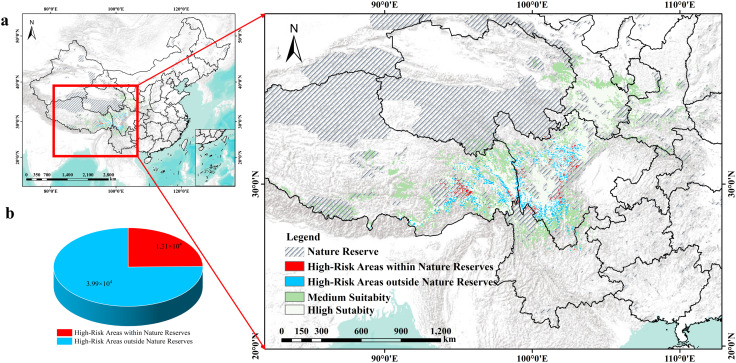
Distribution of medium- and high-habitat suitability areas and high-risk zones for *S. hexandrum.* **(A)** Overlay of high-risk zones and medium-/high-suitability areas with nature reserves. **(B)** Proportion of high-risk zones located within the nature reserve network.

To analyze the range and type of drivers for the spatial differentiation of *S. hexandrum*’s suitable areas, the Risk Detector of the Optimal Parameters Geographical Detector was employed. The results (**Supplementary Table 2**) indicate that high-risk intervals for annual precipitation (bio12) are 574–1070 mm, for precipitation of the driest month (bio14) are 2–13 mm, and for precipitation seasonality (bio15) are 76.8-96.7%. Among temperature-related factors, high-risk intervals for isothermality (bio3) are 38.4-48.8, for temperature seasonality (bio4) are 277-719, and for minimum temperature of the coldest month (bio6) are -19.1 to -1.54 °C. In terms of topographical factors, the high-risk interval for altitude is 2570–4690 m, and for slope (slope) is 13.6-49.9°. To further delineate high-risk distribution areas for *S. hexandrum*, the risk intervals of these environmental factors were spatially overlaid to identify areas simultaneously meeting all risk thresholds, referred to as high-risk areas (**Figure 9A**). The results show that high-risk areas are primarily concentrated in central and western Sichuan and eastern Tibet, exhibiting a distinct spatial clustering characteristic. The environmental conditions in these areas align with the optimal range for *S. hexandrum* growth and are located within the moderate to high suitability areas predicted by the MaxEnt model, representing core regions of its potential distribution.

To assess the current conservation status of *S. hexandrum*’s high-risk areas, a spatial overlay analysis was conducted between the identified high-risk areas and existing nature reserves. The area and proportion of high-risk areas inside and outside nature reserves were calculated using ArcGIS spatial analysis tools (**Figure 9B**). The results indicate that a significant portion of the high-risk areas for *S. hexandrum* lie outside nature reserves (3.99 × 10^4^ km^2^), with only a small fraction (1.31 × 10^4^ km^2^) covered by existing nature reserves. This finding suggests that the current nature reserve network provides insufficient protection for the high-risk areas of *S. hexandrum*, leaving a substantial amount of potential suitable habitat unprotected.

#### 3.7.3 Ecological detection analysis

The influence of any two factors showed statistically significant differences. The Ecological Detector was used to assess the significance of the influence of driving factors on the spatial differentiation of *S. hexandrum’s* suitable areas. Ecological detection results (**Supplementary Figure 3**) indicate that the influence of each environmental factor on the spatial differentiation of *S. hexandrum’s* suitable areas is significantly different (P< 0.05). Significant differences (P< 0.05) were observed between pairs of factors including slope (slope), altitude (elev), minimum temperature of the coldest month (bio6), temperature seasonality (bio4), isothermality (bio3), precipitation seasonality (bio15), precipitation of the driest month (bio14), and annual precipitation (bio12). This suggests that different environmental factors play unique roles and exert varying levels of influence in shaping the spatial distribution patterns of *S. hexandrum*. Each factor contributes heterogeneously to the formation of suitable habitats, and no single factor can fully explain its spatial differentiation.

### 3.8 Dynamic distribution characteristics of *S. hexandrum’s* suitable areas under future climate change scenarios

**Figure 10:**
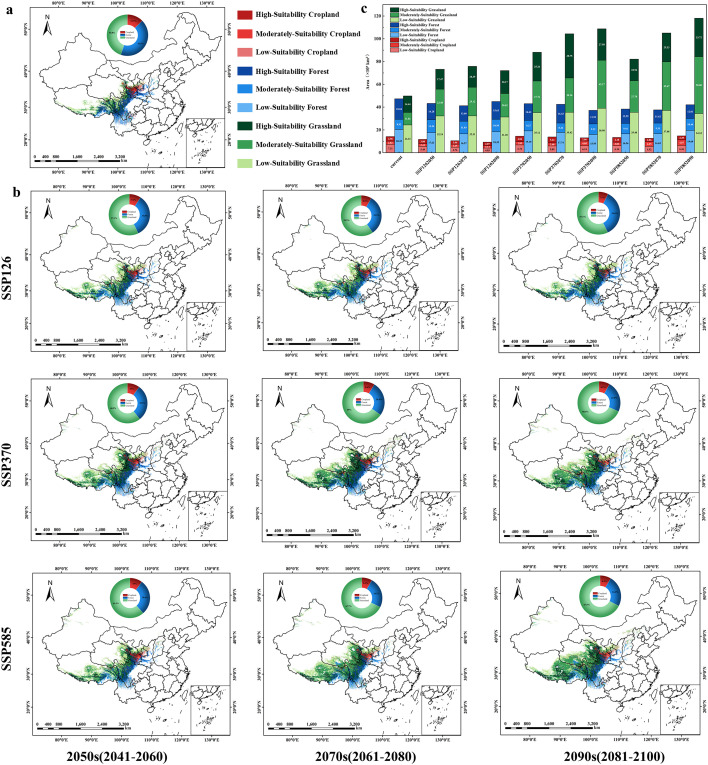
Distribution of suitable habitats for *S. hexandrum* under land use change. **(A)** Land use distribution within the suitable habitats under the current climate scenario. **(B)** Land use distribution within the suitable habitats under a future climate scenario. **(C)** Area (×10^4^ km^2^) of land use types within the suitable habitats under the future climate scenario.

Under current climate conditions, the land use types within the suitable areas of *S. hexandrum* exhibit distinct spatial differentiation (**Figures 10A, C**). Suitable areas are primarily concentrated in southwestern China (Sichuan, Gansu, Yunnan, Tibet, etc.). Grassland is the most prevalent suitable land use type (totaling approximately 4.979 × 10^5^ km^2^), followed by forest land (approximately 4.731 × 10^5^ km^2^) and cropland (approximately 1.411 × 10^5^ km^2^). Spatially, suitable cropland areas are mainly found in low-altitude agricultural zones, grassland suitable areas are widely distributed along the eastern edge of the Qinghai-Tibet Plateau and in the southwestern mountainous regions, and forest land suitable areas are concentrated in the Hengduan Mountains and surrounding forested regions.

Under future climate change scenarios, the suitable areas of *S. hexandrum* show a significant expansion trend, with the magnitude of change closely correlated with emission scenarios (**Figures 10B, C**). Moderately suitable grassland areas continue to increase, reaching 4.217 × 10^5^ km^2^ and 5.000 × 10^5^ km^2^ by the 2090s under SSP370 and SSP585 scenarios, respectively. Changes in forest and moderately to highly suitable areas show relative stability, with an overall decreasing trend in area. Highly suitable forest land reaches its minimum at 1.162 × 10^5^ km^2^ by the 2070s under the SSP585 scenario, while moderately suitable forest land remains between 9-11 × 10^4^ km^2^ under most scenarios. Moderately to highly suitable cropland areas exhibit complex fluctuations; under the SSP585 scenario in the 2090s, moderately suitable cropland increases to 4.87 × 10^4^ km^2^, but highly suitable cropland areas generally show a declining trend. This indicates that grassland ecosystems will become the primary carriers of moderately to highly suitable *S. hexandrum* under future climate warming, followed by forest land, while the conservation value of moderately to highly suitable cropland warrants particular attention. In terms of temporal evolution, the period before the 2050s is considered an adjustment phase, followed by a rapid change phase from the 2050s to the 2070s, with expansion towards higher altitudes and latitudes, and stabilization occurring after the 2070s. This suggests that forest and grassland ecosystems will be the most significant potential distribution areas for *S. hexandrum* in the future.

## 4 Discussion

### 4.1 Spatial pattern of potential suitable areas and the impact of climate change on *S. hexandrum*

The optimized MaxEnt model used in this study revealed that the potential distribution of *S. hexandrum* in China was primarily concentrated in the eastern Qinghai-Tibet Plateau and the Hengduan Mountains region. This distribution pattern was consistent with the species’ ecological adaptation to high-altitude and low-temperature environments (Wani et al., 2025). The mechanisms driving future range shifts are mainly governed by temperature and precipitation thresholds: as global warming progresses, areas that were previously too cold (e.g., minimum temperature of the coldest month below -18 °C) become climatically suitable, while lower-altitude regions may exceed the species’ thermal tolerance. This may lead to systematic contraction at warm-range margins and expansion into previously uninhabited high-altitude and high-latitude areas. Current suitable areas showed a spatial distribution centered on Sichuan, Tibet, and Gansu, with a total area of 1.1608 × 10^6^ km^2^. The process of range shift under climate change may involves three phases: an initial lag phase where dispersal is limited by seed availability and habitat connectivity, an expansion phase where newly suitable areas are colonized, and a potential stabilization phase if climate stabilizes. Our results indicated that expansion was modest before the 2050s, increased rapidly between the 2050s and 2070s, and then relatively stabilized after the 2070s. This pattern may result from the combined effects of topography, climate, and historical factors. For instance, studies on various plants in the Qinling-Qinghai-Tibet Plateau region, such as *Sabina tibetica*, have found that their suitable areas are closely associated with terrain and climate factors and are predominantly located in mountainous and plateau regions (Li et al., 2025). The impact of climate change showed clear scenario dependence. Under low emission scenarios (SSP126), suitable habitats remained relatively stable, with niche overlap maintained above 0.84-0.89. However, under moderate to high emission scenarios (SSP370 and SSP585), suitable habitats are projected to expand significantly by the 2090s, with areas increasing by 45.14% and 61.70%, respectively, and the habitat centroid shifting northwestward by 298.39 km and 333.74 km. This northwestward expansion is driven may be driven by rising temperatures create novel thermal niches at higher elevations and latitudes, while precipitation patterns (bio12: 600–700 mm optimal range) remain suitable in these regions. The species’ cold tolerance (minimum temperature of the coldest month between -18 °C and -3 °C) may allow it to track favorable isotherms upward. This expansion pattern is consistent with the general trend of high-altitude plants migrating to higher altitudes and latitudes in response to global warming, although rapid expansion may exceed the species’ natural dispersal capabilities, leading to a lag in actual distribution (Ma et al., 2022). Further MESS analysis indicated significant differences in precipitation seasonality (Bio15) and mean temperature of the wettest quarter (Bio8) between future and current regions with high similarity, suggesting that novel climatic conditions in newly suitable areas may impose physiological stress and exclude populations unable to cope with altered precipitation regimes (Mishra et al., 2023). Notably, ecological niche overlap decreased significantly in the 2070s across all scenarios, indicating that this period may represent a critical turning point for climate change impacts and should receive special consideration in conservation strategies.

### 4.2 Limitations of key environmental variables on the spatial distribution of *S. hexandrum* and driving factors

Through synergistic analysis of MaxEnt and the Optimal Parameters Geographical Detector, the environmental driving mechanisms of *S. hexandrum* were systematically elucidated. Altitude, minimum temperature of the coldest month, and annual precipitation accounted for 81.6% of the cumulative contribution. However, the explanatory power of any single factor was relatively low, with the maximum q-value being only 0.245 for altitude. This finding highlights the limited ability of individual environmental gradients to explain species distribution and underscores the necessity of analyzing factor interactions. In contrast, the interaction effects, such as bio12 ∩ elev (q=0.685) and bio12 ∩ bio4 (q=0.675), were significantly stronger than any single factor, confirming that the distribution of *S. hexandrum* is primarily controlled by the synergistic regulation of hydrothermal conditions, rather than by a single environmental gradient (Secrafi et al., 2025). Altitude, as the most influential factor (34.5% contribution), exerts its effects by indirectly regulating temperature, humidity, and radiation through multiple factors (Sun et al., 2024). Response curves showed a peak in occurrence probability for *S. hexandrum* between 2800–3500 m, with a sharp decline in suitability at excessively high or low altitudes, indicating a non-linear response and a strict requirement for microenvironments within specific altitudinal belts. This aligns with findings from studies on soil organic carbon distribution in mountainous forests, where altitude significantly influences climate and vegetation patterns, thereby affecting ecosystem processes (Suleymanov et al., 2024). The adaptability to minimum temperature of the coldest month between -18 °C and -3 °C suggests a certain degree of cold tolerance, with an optimal temperature threshold around -10 °C providing valuable reference for predicting distribution changes under future warming conditions (Chai et al., 2025). Annual precipitation exhibits an optimal range of 600–700 mm, a relatively narrow window that explains why *S. hexandrum* cannot be widely distributed in areas with abundant precipitation in the subtropics or arid conditions of the northwest inland (Tian et al., 2025). The significance of temperature seasonality (bio4,16.8% contribution) reflects physiological adaptation to the rhythm of temperature fluctuations, and moderate seasonal variations may serve as essential environmental cues for completing the species’ life cycle. Based on the principles of selecting medicine based on location and manufacturing medicine based on location, establishing standardized cultivation bases in suitable areas of western Sichuan and southern Gansu, which meet the ecological requirements of *S. hexandrum* (altitude 2800–3500 m, annual precipitation 600–700 mm), can both satisfy market demand and preserve the integrity of the wild gene pool.

Notably, the contribution rate of precipitation of the driest month is only 0.4%, whereas its permutation importance reaches 18.4%. This discrepancy suggests that this factor may play a critical ecological filtering role under extreme conditions, leading to species disappearance below a certain threshold, while having limited impact on the overall distribution pattern within the suitable range. The low contribution rate of topographical factors (slope and aspect, less than 1%) indicates a weak direct influence, but they indirectly affect species distribution by altering local hydrothermal redistribution, with their effects integrated into the variation of altitude and climatic factors, underscoring the necessity of considering both direct and indirect factors (Yin et al., 2024).

### 4.3 Habitat conservation and refined management of *S. hexandrum*

Risk detection identified high-risk areas (5.30 × 10^4^ km^2^) that were primarily concentrated in central and western Sichuan and eastern Tibet, which should be prioritized for *in-situ* conservation. However, the finding that 75.3% of these areas are located outside existing nature reserves highlights the inadequacy of the current conservation network in addressing the microhabitat requirements of endangered medicinal plants (Gao et al., 2025). This likely reflects the fact that nature reserves are often designated for flagship species, scenic landscapes, or broader ecosystem representation, rather than to target the specific microhabitats (such as particular altitudinal ranges, slope aspects, or understory conditions) required by an understory medicinal plant like *S. hexandrum*. Differentiated strategies are recommended: strict monitoring and habitat maintenance within protected areas, and flexible approaches such as community-managed conservation areas for areas, for sites outside existing reserves. High-risk areas in central Sichuan, which are core distribution areas and traditional medicinal herb production regions, face significant pressure from human harvesting. Therefore, monitoring and early warning mechanisms, along with a permit system for harvesting, are urgently needed. This approach is consistent with Liu et al.’s concept of “monitoring and early warning zones” in the ecological risk assessment of geohazards in Natural World Heritage Sites (Liu et al., 2019).

Expected changes in land-use patterns play a critical role in shaping future range shifts. Our analysis indicates that grasslands (4.979 × 10^5^ km^2^) and forests (4.731 × 10^5^ km^2^) are the primary carrier ecosystems for endangered medicinal plants (Guan et al., 2024). Under future climate scenarios, moderately suitable grassland areas are projected to continue increasing, while highly suitable forest areas are expected to decrease. This divergence arises because grasslands, being more open and less structurally complex, allow for faster plant migration and establishment under changing climates, whereas forests (especially old-growth forests) may act as barriers or experience time lags in species turnover. The process of range shift is therefore mediated by land-use type: grassland ecosystems facilitate expansion, while croplands may act as ecological traps due to agricultural disturbances, and forests provide stable but slowly changing habitats. Consequently, grassland ecosystem conservation becomes critically important for maintaining population stability (Li and Zhou, 2025). An ecosystem-based conservation strategy that prioritizes the entire ecosystem over single-species protection should be adopted. Maintaining the integrity and connectivity of grasslands could simultaneously benefit multiple alpine medicinal plants. Although the area of suitable cropland (1.411 × 10^5^ km^2^) is smaller, highly suitable cropland areas are show a declining trend. This decline may be driven by agricultural intensification and land conversion, which fragment suitable patches and reduce habitat quality. Therefore, establishing ecological refuges for *S. hexandrum* in agro-pastoral mosaic areas through eco-friendly agricultural practices is recommended, which is consistent with Lee and Liu’s research on the synergistic benefits of urban farmlands as a climate change adaptation strategy (Lee and Liu, 2023). Considering the observed northwestward migration trend of *S. hexandrum’s* suitable areas’ centroid under future climate scenarios, climate-adaptive conservation corridors should be established in future high-suitability areas, such as southern Gansu and eastern Qinghai. Wang et al.’s research also explored climate-adaptive migration corridors for various species in China, emphasizing their importance in biodiversity conservation (Wang et al., 2025).

### 4.4 Conservation and sustainable development strategies for endangered medicinal plants

The conservation and sustainable development of endangered medicinal plants represent a global challenge, particularly with the continuous growth in demand for traditional medicines (Chen et al., 2016). Using *S. hexandrum* as an example, its conservation predicament profoundly reflects the inherent conflict between medicinal plant utilization and the limitations of wild resources. Over-harvesting is a direct cause, but the fundamental issue lies in the irreconcilable tension between increasing medicinal demand and finite wild resources. Merely restricting harvesting is insufficient for long-term conservation; a comprehensive conservation-propagation-substitution strategy must be established (Kaur et al., 2022). The five dimensions of traditional Chinese medicine geography—ecological attributes, temporal attributes, social attributes, economic attributes, and regional attributes—provide a complete framework for developing integrated conservation strategies: ecological attribute research clarifies habitat requirements, temporal attribute research analyzes patterns of production area changes, social and economic attribute research optimizes resource allocation, and regional attribute research achieves spatial balance between conservation and utilization (Zhang et al., 2025c). This integrated strategy, based on the medicine-geography relationship, organically combines conservation-propagation-substitution, offering a scientific pathway for the sustainable utilization of endangered medicinal plant resources and holding significant theoretical and practical value for the construction of the entire traditional medicine resource conservation system.

### 4.5 Limitations and future outlook

Despite serving as a valuable model for endangered medicinal plant conservation and sustainable utilization, this study has several limitations. Firstly, the model relies on the assumption of ecological niche conservatism, which implies that the species’ environmental tolerances remain constant over time. This assumption may not hold under rapid climate change, as species could undergo adaptive evolution or ecological niche shifts (e.g., through phenotypic plasticity or genetic adaptation). The current model also does not account for the species’ rapid adaptive capacity or the impact of interspecific interactions (e.g., pollinators, pathogens) on its distribution. Future research should integrate population genetics and community ecology data to construct more realistic predictive models. Secondly, as acknowledged in the Methods section, this study used a single GCM (BCC-CSM2-MR), which may not capture the full range of future climate uncertainty; a multi-model ensemble approach would improve robustness. Thirdly, the 2.5′ resolution of climate data may not accurately capture local microclimates in complex terrain. Alpine plants’ actual distribution is strongly influenced by microtopography, microclimate, and soil heterogeneity; therefore, higher resolution data should be incorporated to improve prediction accuracy. Fourthly, while this study employed the widely used and highly performant MaxEnt model, future research should consider ensemble modeling approaches, combining outputs from multiple models, to enhance the accuracy of predicting endangered medicinal plant habitat distribution under climate change.

Future research should expand from single-species conservation to the community level of alpine medicinal plants, identifying biodiversity hotspots to achieve synergistic conservation for multiple species. Additionally, research should focus on the impact of climate change on the content of active compounds and medicinal efficacy of medicinal plants, as even if the species can survive in new environments, its medicinal value may change, which is crucial for sustainable utilization strategies. These extended studies will provide a solid scientific foundation for building a more comprehensive and effective conservation system for endangered medicinal plants.

## 5 Conclusion

Addressing the scientific need for endangered medicinal plant conservation amidst climate and land-use change, this study established a synergistic analytical framework combining an optimized MaxEnt model with an Optimal Parameters Geographical Detector(OPGD). This framework systematically evaluated the habitat suitability patterns, driving mechanisms, and future distribution trends of the endangered medicinal plant *S. hexandrum*. The study confirmed that *S. hexandrum’s* current distribution is concentrated in the eastern Qinghai-Tibet Plateau, and future climate warming is expected to drive significant expansion towards the northwest alpine regions, with the 2070s identified as a critical turning point. The driving mechanism analysis moved beyond traditional single-factor explanations, revealing the synergistic regulatory role of hydrothermal coupling processes on species distribution and highlighting that the explanatory power of environmental factor interactions significantly surpasses that of individual factors, thereby deepening the understanding of the ecological adaptation mechanisms of alpine medicinal plants. The identification of conservation gaps indicated that the current conservation network inadequately considers the microhabitat requirements of endangered medicinal plants, with over 75% of high-risk areas located outside protected areas. Land-use analysis underscored the strategic importance of protecting grassland and forest ecosystems, providing a scientific basis for ecosystem-based conservation strategies. The comprehensive assessment framework and identified priority conservation areas established by this study offer precise spatial guidance for conservation planning, climate-adaptive management, and sustainable utilization of endangered medicinal plants, holding significant reference value for the construction of a medicinal plant resource conservation system within the context of global change. Future research should extend from single-species to community-level conservation, integrating population genetics, high-resolution microclimate data, and monitoring of active compound content, to develop a more comprehensive conservation-propagation-substitution strategy. This will provide scientific support for the effective conservation and sustainable utilization of endangered medicinal plant resources.

## Data Availability

The original contributions presented in the study are included in the article/**Supplementary Material**. Further inquiries can be directed to the corresponding author.
