# Supplementary material for: Habitat suitability and driving factors of the endangered medicinal plant Sinopodophyllum hexandrum under climate and land use change
Source: Front Plant Sci. 2026 Jun 3;17:1809149. doi: 10.3389/fpls.2026.1809149 (PMC13272479; doi:10.3389/fpls.2026.1809149)
Supplement: Supplementary file 1 [file DataSheet1.docx]

Table S1. Variables used in the model predictions.

| Variable description | Abbreviated name | Unit |
| --- | --- | --- |
| Slope aspect | aspect | ° |
| Altitude | elev | m |
| Slope gradient | slope | ° |
| Isothermality | bio3 | 1 |
| Temperature Seasonality | bio4 | 1 |
| Min Temperature of Coldest Month | bio6 | ℃ |
| Annual Precipitation | bio12 | mm |
| Precipitation of Driest Month | bio14 | mm |
| Precipitation Seasonality | bio15 | mm |

Table S2. Identification of high-risk areas of driving factors

| Variable | High-risk area |
| --- | --- |
| bio12 | (574，1070] |
| bio14 | (2，13] |
| bio15 | (76.8，96.7] |
| bio3 | (38.4，48.8] |
| bio4 | (277，719] |
| bio6 | (-19.1，-1.54] |
| elev | (2570，4690] |
| slope | (13.6，49.9] |


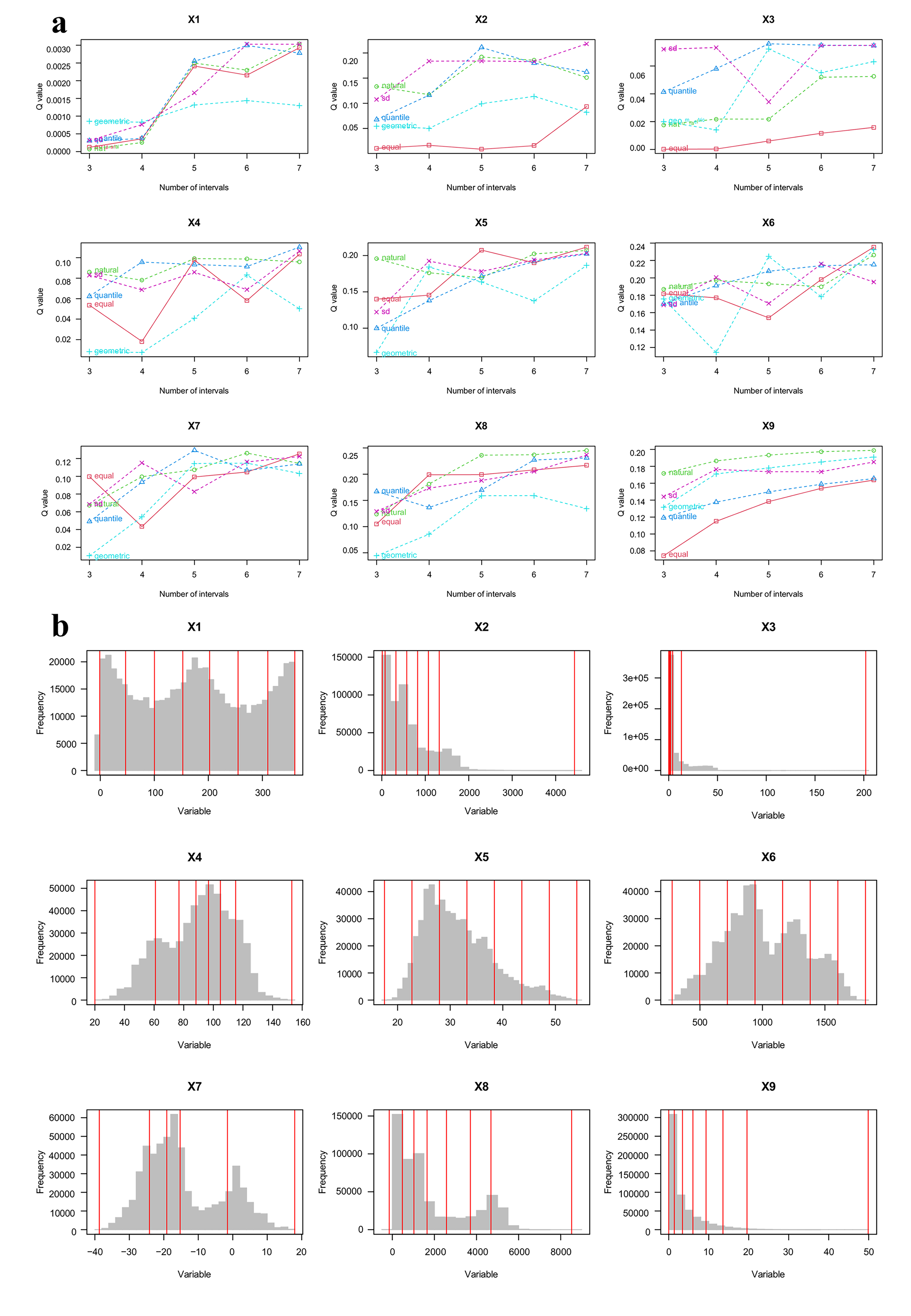


Fig S1. Discretization process and results of the explanatory variables (X1: Aspect, X2: Bio12, X3: Bio14, X4: Bio15, X5: Bio3, X6: Bio4, X7: Bio6, X8: Elevation, X9: Slope).


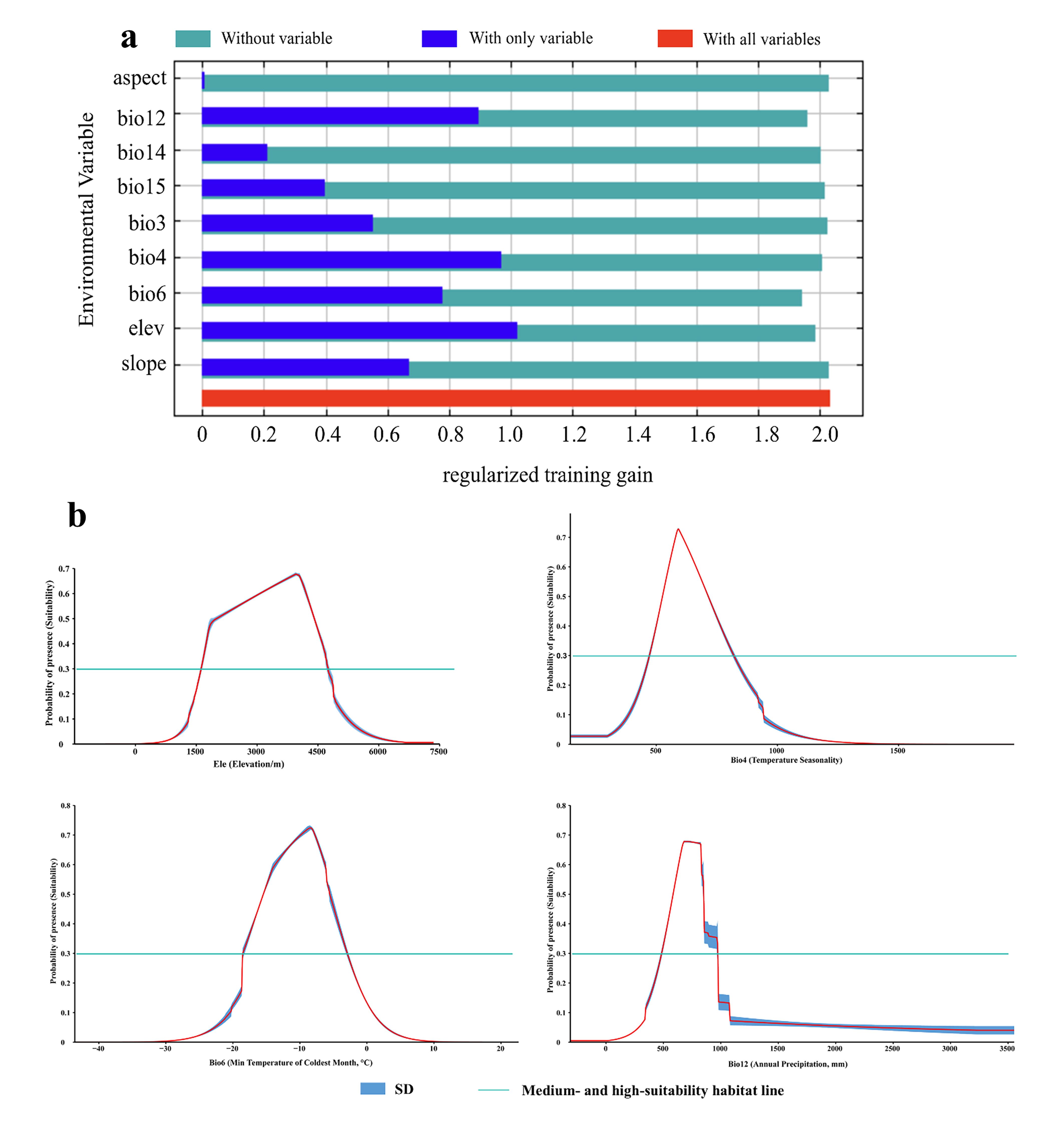


Fig S2. Jackknife test of variable importance (a) and response curves of key environmental variables (b) for *S. hexandrum.*


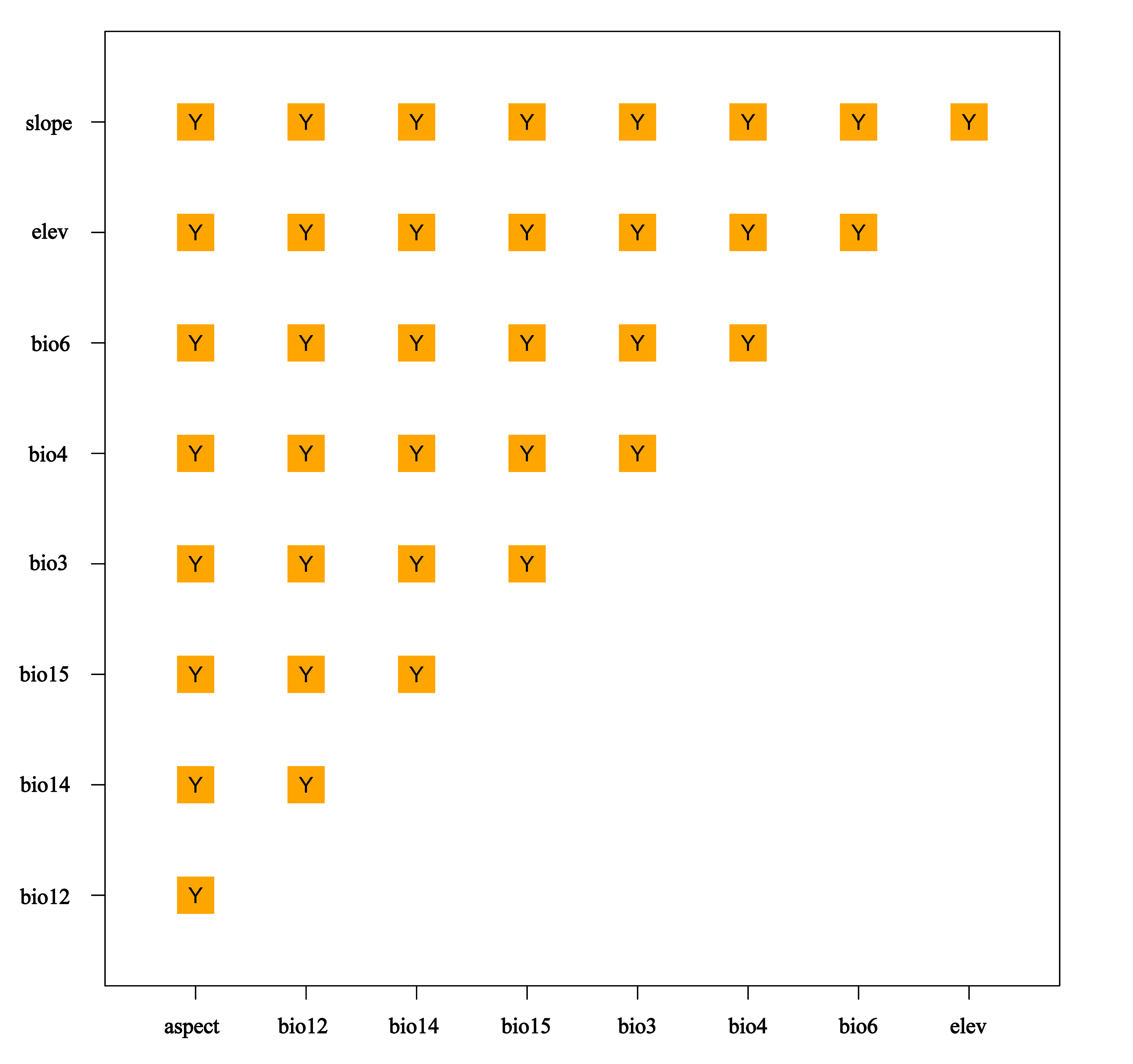


Fig S3. Results of ecological detection (Y indicates a significant difference, N indicates no significant difference).
